# Effect of methylene blue infusion into the inferior mesenteric artery on lymph node retrieval after transabdominal total mesorectal excision for rectal cancer: a retrospective cohort study

**DOI:** 10.3389/fonc.2026.1772591

**Published:** 2026-04-13

**Authors:** Meng Zhao, Rong Zhao, Fusheng Wang, Zhiyong Yang, Deli Chen, Lugen Zuo, Sitang Ge

**Affiliations:** 1 The Fifth People's Hospital of Bengbu, Bengbu, Anhui, China; 2 Department of Gastrointestinal Surgery, The First Affiliated Hospital of Bengbu Medical University, Bengbu, Anhui, China; 3 Bengbu First People's Hospital, Bengbu, Anhui, China

**Keywords:** heparin sodium, lymph nodes, methylene blue, neoplasm staging, rectal neoplasms

## Abstract

**Background:**

This study aimed to investigate the effect of methylene blue injection via the inferior mesenteric artery on lymph node retrieval in postoperative rectal cancer specimens.

**Materials and methods:**

This retrospective cohort study enrolled 120 patients undergoing radical rectal cancer resection by the same surgical team at a hospital in Bengbu City between July 2023 and December 2024. Among these, 60 patients operated on between April and December 2024 underwent heparinization of the surgical specimen post-extraction, followed by methylene blue arterial perfusion (experimental group). The remaining 60 patients, operated on between July 2023 and March 2024, received no special treatment after specimen removal (control group). The two groups were compared regarding total postoperative lymph node detection rates, positive lymph node detection rates, and average time per lymph node examined.

**Results:**

The experimental group exhibited a higher total number of lymph nodes detected and a higher total number of negative lymph nodes detected compared to the control group, with both differences being statistically significant [27.783 ± 9.243 vs. 15.317 ± 7.480, *t* = 8.122, *P* < 0.001; (25.700 ± 9.786) vs. (14.200 ± 7.841), *t* = 7.103, *P* < 0.001]; The number of positive lymph nodes detected in the experimental group increased compared to the control group, but the lymph node metastasis rate decreased, with no statistically significant differences [2.083 ± 3.933 vs. 1.117 ± 2.263, *t* = 1.650, *P* = 0.102; (0.079 ± 0.151) versus (0.090 ± 0.173), *t* = 0.347, *P* = 0.729]; The experimental group exhibited a statistically significant reduction in the average time per lymph node examination compared to the control group [0.785 ± 0.372 min versus 1.632 ± 0.884 min, *t* = 6.839, *P* < 0.001].

**Conclusions:**

Methylene blue injection via the inferior mesenteric artery enhances the total number of lymph nodes detected post-rectal cancer surgery while reducing the average time per lymph node examination. This ensures accurate pathological staging and holds promise for providing more precise evidence for post-operative prognosis assessment and treatment planning in rectal cancer.

## 1 Introduction

Colorectal cancer ranks as the third most common malignant tumor globally, with its incidence rising annually. Lymph node metastasis constitutes the primary route of metastasis in rectal cancer, with patients exhibiting lymph node involvement typically presenting a poorer prognosis (1, 2). Radical surgical resection remains the primary treatment modality (3, 4), and the number of lymph nodes detected in postoperative specimens directly influences the reliability of tumor pathological staging. This has direct implications for determining the necessity of adjuvant radiotherapy and chemotherapy, as well as for further assessing patient prognosis (5–7). The National Comprehensive Cancer Network (NCCN) recommends that patients who have not undergone neoadjuvant therapy should, in principle, have no fewer than 12 lymph nodes examined postoperatively (8). However, pathologists frequently encounter issues such as insufficient lymph node sampling, omission of smaller nodes, or inaccurate lymph node grouping during examination (9). Research indicates that a higher number of lymph nodes examined and more accurate pathological staging correlate with improved patient prognosis (10, 11). Numerous methods have been attempted to obtain a greater number of accurately grouped lymph nodes, but these have not gained widespread adoption due to complex technical procedures or high costs (12, 13). The technique of injecting methylene blue into excised specimens to enhance lymph node retrieval was first proposed by Märkl et al. in 2007 (14).

Many scholars have also confirmed its feasibility (15–18), however, this method has not yet significantly increased the number of lymph nodes retrieved, nor has it significantly improved the retrieval rate for lymph nodes smaller than 5 mm in diameter. This study builds upon that technique with modifications. Specimens are first heparinized to prevent thrombus formation and vascular occlusion. Methylene blue is then injected via the inferior mesenteric artery to trace lymphatic pathways, visualizing lymph nodes along vascular courses under illumination. This approach substantially increases the total number of retrieved lymph nodes and facilitates detection of smaller, more numerous nodes.

## 2 Materials and methods

### 2.1 Patient selection and data collection

This retrospective cohort study employed the following inclusion criteria (1): patients with a confirmed diagnosis of primary rectal cancer without distant metastasis, meeting the criteria for transanal total mesorectal excision (TME) surgery as outlined in the NCCN guidelines for mid-to-low rectal cancer (2); patients with complete clinical case records; (3) standard transabdominal total mesorectal excision for rectal cancer; (4) None of the patients received neoadjuvant chemoradiotherapy prior to surgery, and no concomitant malignancies of other tissue origins.

According to the above criteria, 60 patients who underwent radical resection of rectal cancer by the same treatment group in the Department of Gastrointestinal Surgery of the First Affiliated Hospital of Bengbu Medical University from April 2024 to December 2024 composed the experimental group (MB group). After the surgical samples were isolated, heparinization was performed first, and then methylene blue artery perfusion was performed. With the same inclusion criteria, 60 patients who underwent surgery from July 2023 to March 2024 composed the control group, and no special treatment was given after the surgical samples were isolated. All patients underwent standard transabdominal total mesorectal excision for rectal cancer, which was performed by the same treatment group of doctors. All patients underwent the same preoperative preparation, and postoperative specimens were taken by the same surgeon under a luminescent lamp. There was no significant difference in basic clinical data between the two groups (**Table 1**).

**Table 1:** Patient clinicopathological data.

Variables	MB group(n=60)	Control group(n=60)	P
Age (y)			0.657 ^a^
Mean ± SD	66.4 ± 11.1	65.4 ± 12.3
Sex (n,%)			0.695 ^b^
Female	18(30%)	20(33.3%)
Male	42(70%)	40(66.7%)
BMI (*kg*/*m*^2^)			0.855 ^b^
<24	28(46.7%)	29(48.3%)
≥24	32(53.3%)	31(51.7%)
Tumor size (cm)			0.356 ^b^
<4	28(46.7%)	23(38.3%)
≥4	32(53.3%)	37(61.7%)
Pathomorphologic typing			0.157 ^b^
ulcerative	52(86.7%)	46(76.7%)
else	8 (13.3%)	14(23.3%)
T stage, n (%)			0.307 ^b^
T1-2	14(23.3%)	19(31.7%)
T3-4	46(76.7%)	41(68.3%)
N stage, n (%)			0.306 ^b^
N 0-1	49(81.7%)	53(88.3%)
N 2-3	11(18.3%)	7(11.7%)
vascular invasion			0.831 ^b^
Yes	14(23.3%)	15(25%)
No	46 (76.7%)	45(75%)
neurological violation			0.835 ^b^
Yes	16(26.7%)	15(25%)
No	44(73.3%)	45(75%)
cancer nodule			0.717 ^c^
Yes	3(5.0%)	5(8.3%)
No	57(95.0%)	55(91.7%)
Operative part length (cm)			0.203 ^a^
Mean ± SD	15.550 ± 2.368	15.067 ± 1.716	

^a^t test, ^b^χ2 test, ^c^Fisher's exact probability test.

### 2.2 Methods of specimen processing

Postoperative lymph node dissection was performed according to the 2022 CSCO guidelines for the diagnosis and treatment of colorectal cancer.

#### 2.2.1 Procedure for the experimental group

**Figure 1:**
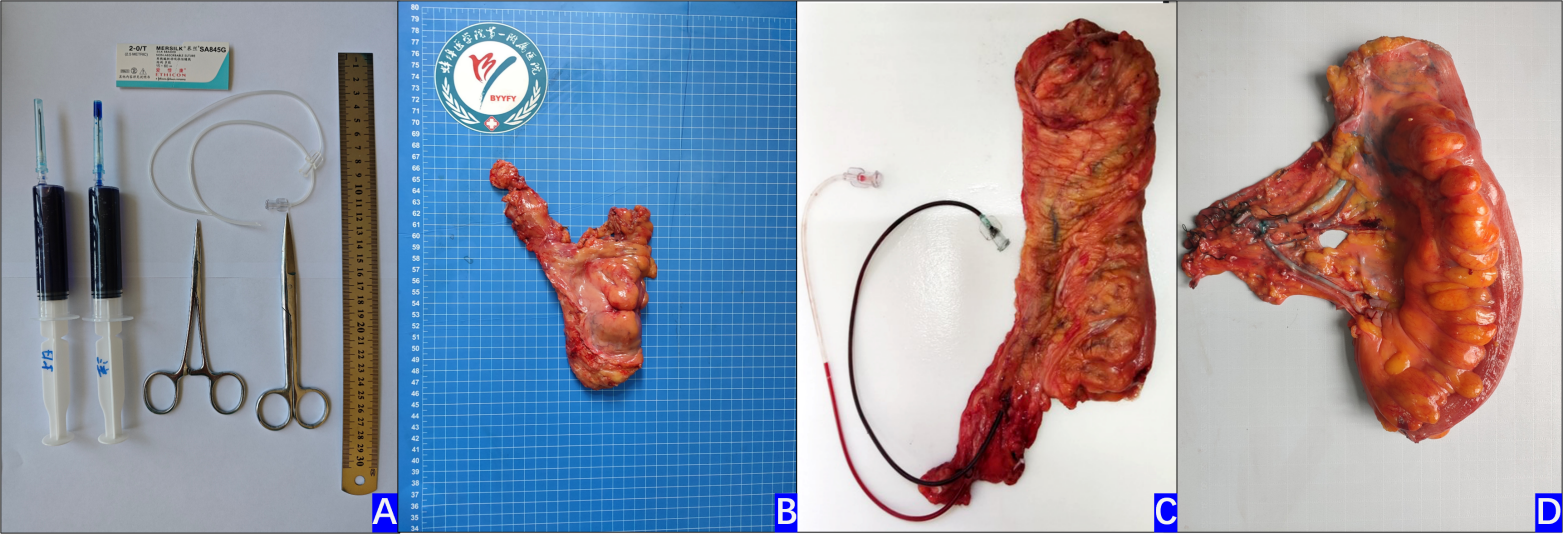
Lymph node retrieval procedure.

**Figure 2:**
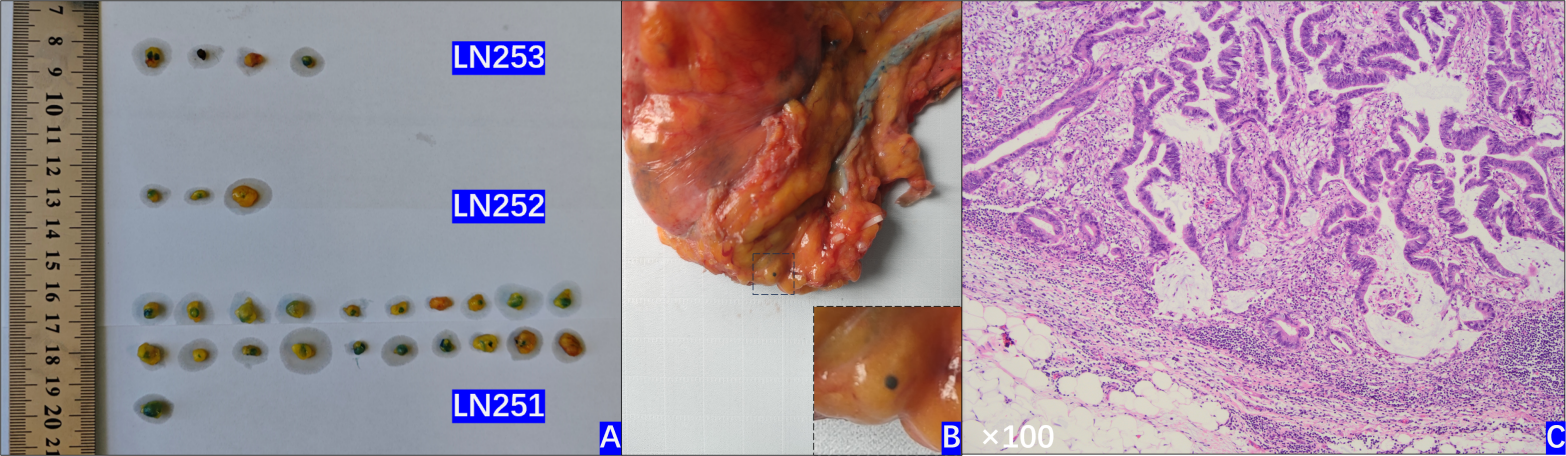
Lymph nodes after sampling.

The MB group were as follows: ① Prepare all relevant items preoperatively. ② Within 30 minutes of the rectal cancer specimen being removed from the body, clean off bloodstains, photograph, measure, and retain images. ③ Reconfirm vascular transection status with the operating surgeon and place the specimen on the examination table. ④ Locate the inferior mesenteric artery. Incise 1.5 cm along the vessel course to expose the mesentery. Insert a scalp needle (intravenous cannula) into the inferior mesenteric artery, securing it with silk ligature to prevent dislodgement. Employ the same technique to insert another scalp needle into the inferior mesenteric vein. ⑤ Slowly inject 4ml of 0.01mg/L low molecular weight heparin sodium mixed with 15ml methylene blue solution [5ml methylene blue (1mg/ml, Jichuan Pharmaceutical Group Co., Ltd., 2ml:20mg) + 10ml saline] into the inferior mesenteric artery. Methylene blue solution will be observed flowing out through the inferior mesenteric vein. Clamp the inferior mesenteric vein. Slowly inject a further 15 ml of methylene blue solution (5 ml of 1 mg/ml methylene blue + 10 ml saline) into the inferior mesenteric artery. ⑥ After 10 minutes, remove the scalp needle. Position the specimen under the luminescent lamp and adjust its brightness. Dissect the mesentery along the vascular course to sequentially harvest lymph nodes from groups 253, 252, and 251 for examination (**Figure 1**). ⑦ Measure the size of the harvested lymph nodes, place them individually in 10% formalin solution with clear labelling, and send to the pathology department (**Figure 2**). ⑧ Pathologists examine the specimens and relevant lymph nodes. The pathology report specifies the number of lymph nodes sampled per group and the number of positive metastatic lymph nodes.

#### 2.2.2 Procedure for the control group

In the control group: ① Within 30 minutes of removal from the body, clean bloodstains from the rectal cancer surgical specimen, photograph it, and record measurements. ② Position the specimen under a light source, adjust brightness, and dissect along vascular courses to sequentially harvest lymph nodes from groups 253, 252, and 251 for examination. ③ Place harvested lymph nodes into 10% formalin solution, label them, and submit to the pathology department. ④ Pathologists shall examine the specimen and relevant lymph nodes. The pathology report shall specify the number of lymph nodes sampled from each group and the number of positive metastatic lymph nodes.

### 2.3 Statistical analysis

The total number of lymph nodes harvested, the number of negative lymph nodes harvested, the ratio of the number of positive lymph nodes to the total number of lymph nodes harvested (lymph node metastasis rate), the number of small lymph nodes (<5 mm) and the average time of each lymph node harvested were compared between the two groups. All the parameters were analyzed via SPSS 25.0 software. Continuous data are expressed as the means ± SDs, and count data were analyzed via the chi-square test or Fisher’s exact probability test. The measurement data were normally distributed. P < 0.05 was considered statistically significant.

## 3 Results

The experimental group examined a total of 1,667 lymph nodes, significantly exceeding the 919 examined in the control group. Compared with the control group, the experimental group exhibited significantly higher total lymph node detection rates and negative lymph node detection rates, with statistically significant differences [(27.783 ± 9.243) nodes versus (15.317 ± 7.480) nodes, *t* = 8.122, *P* < 0.001; (25.700 ± 9.786) vs. (14.200 ± 7.841), *t* = 7.103, *P* < 0.001]; The number of positive lymph nodes detected in the experimental group increased compared to the control group, but the lymph node metastasis rate decreased, with no statistically significant differences [(2.083 ± 3.933) vs. (1.117 ± 2.263), *t* = 1.650, *P* = 0.102; (0.079 ± 0.151) versus (0.090 ± 0.173), *t* = 0.347, *P* = 0.729]; Compared with the control group, the experimental group exhibited a statistically significant reduction in the average time per lymph node biopsy: [(0.785 ± 0.372) min versus (1.632 ± 0.884) min, *t* = 6.839, *P* < 0.001] (**Table 2**).

**Table 2:** Comparison of lymph node detection between the two groups.

Clinical information	MB group (n=60)	Control group (n=60)	P	T
Average number of LN harvest	27.783 ± 9.243	15.317 ± 7.480	<0.001^a^	8.122
The number of negative LN	25.700 ± 9.786	14.200 ± 7.841	<0.001^a^	7.103
Average number of metastatic LN	2.083 ± 3.933	1.117 ± 2.263	0.102 ^a^	1.650
Average time per lymph node retrieval(min)	0.785 ± 0.372	1.632 ± 0.884	<0.001 ^a^	6.839
Lymph node metastasis rate	0.079 ± 0.151	0.090 ± 0.173	0.729 ^a^	0.347
Proportion with <12 nodes examined, per total number of lymph nodes(%)	3.3(2/60)	26.7(16/60)	0.001^c^	
Rate of small lymph nodes (<5 mm) in total count (%)	51.6	26.7		

^a^t test, ^b^χ2 test, ^c^Fisher's exact probability test.

Among the experimental group patients, 23 cases developed lymph node metastasis, with one case exhibiting skipped metastasis. The lymph node metastasis rates were 10.13% in group 251, 3.68% in group 252, and 1.69% in group 253. The sorting rate for lymph nodes with diameters <5mm reached 51.60%.

A comparison of our protocol with those of previous key studies demonstrated that heparin pretreatment followed by arterial methylene blue perfusion resulted in a greater number of lymph nodes retrieved (**Table 3**).

**Table 3:** Comparative data from previous studies on [Methylene Blue Staining Techniques.

Study	MB group				Control group LN (mean ± SD)	Country
	The artery used	MB concentration (mg/ml)	Volume (ml)	LN (mean ± SD)		
Märkl B et al., 2007	superior rectal artery	2.5~3.33	15–20	27 ± 7	14 ± 4	Germany
Suszták N et al., 2022	main supplying arteries	1.67	30	14 ± 6	11 ± 8	Hungary
Klepšytė E et al., 2012	inferior mesenteric artery	5	30	18 ± 5	14 ± 6	Lithuania
Hong-Ke Cai et al., 2012	main supplying arteries	2	2	23.8 ± 6.9	12.2 ± 3.2	China
Jianpei Liu et al., 2017	superior rectal artery	0.33	16	23.2 ± 4.7	11.7 ± 3.4	China
G. Kır a et al., 2014	main supplying arteries	2.5	10-20	24.48 ± 12.99	21.49± 13.76	Turkey
This study	inferior mesenteric artery	0.33	30	27.78 ± 9.24	15.32 ± 7.48	China

## 4 Discussion

The number of lymph nodes detected in postoperative specimens from rectal cancer patients influences tumor staging, which in turn affects prognosis and subsequent treatment (19, 20). It also reflects the adequacy of the surgeon’s intraoperative lymph node dissection. The presence of positive lymph nodes particularly indicates the potential for local recurrence (21). If fewer than ten negative lymph nodes are retrieved, chemotherapy is recommended, potentially leading to overtreatment in some patients (22). There is also literature indicating that improving the accuracy of sentinel lymph node detection is highly significant (23). Currently, postoperative lymph node sampling for rectal cancer is typically performed by pathologists. At this stage, specimens undergo prolonged formalin fixation and dehydration, causing the fatty tissue enveloping lymph nodes to become toughened, blood within vessels to coagulate, and lymph node tissue to shrink (24), This frequently results in insufficient lymph node sampling, failure to detect smaller nodes, and impairs pathologists’ ability to accurately assess lymph node grouping and stationing. Maximizing the number of lymph nodes obtained from postoperative rectal cancer specimens is essential for establishing accurate tumor staging and is recommended as a basis for determining patient prognosis and adjuvant chemotherapy decisions (25, 26). Although the optimal number of lymph nodes to be examined post-rectal cancer surgery remains controversial, previous studies indicate that a higher number of detected lymph nodes correlates with improved patient prognosis (27, 28), Moreover, the majority of metastatic lymph nodes are typically smaller than 5 mm (29–31), making them prone to omission during routine examination. To obtain more lymph nodes, various methods have been attempted, such as fat-dissolving techniques and nanocarbon tracing technology. However, these approaches are time-consuming, labor-intensive, and technically demanding, limiting their widespread adoption (13, 14). Methylene blue, an aromatic heterocyclic macromolecular compound, rapidly enters the bloodstream following arterial injection. It subsequently circulates through the bloodstream into the lymphatic vessels, thereby staining lymph nodes.

During our initial experiments, we observed that injecting methylene blue solution directly into arterial vessels yielded suboptimal lymph node staining. Lymph nodes proximate to arterial vessels stained well, whereas those near terminal arterial branches—particularly smaller lymph nodes—displayed poor visualization. Adjusting the concentration failed to produce satisfactory results. Previous studies have also reported that injecting methylene blue solution into the artery does not substantially increase lymph node yield (32, 33). A comparative analysis of findings from key prior studies revealed that (15, 34–36), despite adjustments in methylene blue concentration and dosage by most researchers, the number of lymph nodes retrieved did not significantly improve. Given that our initial experiments also yielded suboptimal results, we hypothesized that this may be attributable to thrombus formation due to blood coagulation, leading to vascular obstruction and preventing the methylene blue solution from adequately entering the circulatory system and staining the lymph nodes. Consequently, the experiment was modified by pre-treating the vessels with low molecular weight heparin sodium, leveraging its anticoagulant properties to prevent thrombus formation (37–39).

We first inject a mixture of low molecular weight heparin sodium and a prepared methylene blue solution into the inferior mesenteric artery, allowing it to flow out via the venous system. This not only prevents thrombus formation from obstructing the pathway but also enables assessment of whether the specimen’s blood circulation remains unimpeded. Subsequently, the inferior mesenteric vein was clamped off. Methylene blue solution was then injected into the specimen via the inferior mesenteric artery, increasing the pressure differential between tissue fluid and lymphatic fluid. Given the small particle size and high diffusivity of methylene blue, facilitating their easy penetration through vascular and lymphatic walls via hemodynamic forces into the lymphatic circulation. These particles are subsequently phagocytosed by macrophages and transported within the lymphatic system, thereby staining lymph nodes. The final results confirmed the author’s hypothesis: pretreatment of vessels with low molecular weight heparin sodium yields a greater number of smaller lymph nodes.

This method not only increases the total number of lymph nodes examined but also yields a higher proportion of lymph nodes smaller than 5 mm in diameter. Furthermore, the average time required to examine each lymph node is reduced. The number of positive lymph nodes detected in the experimental group increased compared to the control group, but the proportion of positive lymph nodes in both groups did not reach statistical significance. The authors believe that the primary effect of methylene blue ex vivo arterial perfusion lies in enhancing the visualization of lymph nodes and lymphatic tissue, thereby increasing the overall detection rate of lymph nodes. This is particularly beneficial for identifying smaller lymph nodes that are difficult to discern macroscopically. Since smaller lymph nodes exhibit higher metastatic potential, an increase in total detection numbers may correspondingly elevate the absolute count of positive lymph nodes. However, methylene blue lacks specific affinity for metastatic lymph nodes; it enhances “detection capability” rather than “metastatic occurrence probability.” Therefore, it is understandable that no significant differences in lymph node metastasis rates or metastasis-positive lymph node ratios were observed when tumor biological characteristics were similar. Furthermore, previous studies suggest that the metastasis-positive lymph node ratio holds certain value in staging assessment and prognosis determination for colorectal cancer (40, 41). By carefully controlling methylene blue concentration—avoiding excessive staining that obscures the entire specimen or insufficient staining that renders blue-stained lymph nodes difficult to locate—blue-stained lymph nodes become more readily identifiable against the yellow fatty background. This minimizes the need for repeated specimen trimming, thereby preserving margins for assessment and tumor sampling while reducing overall specimen damage.

## 5 Conclusion

The innovation of this study lies in the pre-treatment of ex vivo specimens with a methylene blue solution mixed with low molecular weight heparin sodium. This ensures unimpeded blood circulation while preventing excessive methylene blue injection that could cause blue dye leakage. Leveraging methylene blue’s strong penetrating power and favorable staining properties, lymph nodes are stained. Under illumination, dissecting the mesentery along vascular pathways minimizes vascular disruption and tissue contamination, facilitating rapid identification of blue-stained lymph nodes—particularly those under 5mm in diameter. Crucially, methylene blue staining does not interfere with microscopic observation of tumor cells or lymphocytes, rendering this method clinically viable for broader implementation.

### 5.1 Limitations

This retrospective study design presents certain limitations, notably the absence of randomization, including a small sample size and lack of postoperative follow-up information for patients. This study employed a time-series cohort design; since the two groups of patients were not enrolled simultaneously, this design may carry an inherent risk of long-term trend bias. The necessity of heparin pretreatment remains inadequately validated, and this study cannot yet quantify its independent contribution. Future prospective studies or ex vivo controlled experiments comparing different processing strategies—such as “heparinization + methylene blue” versus “methylene blue alone”—are planned to further validate mechanisms and optimize protocols. This study did not perform subgroup analyses stratified by key clinical variables (e.g., tumor staging [T/N staging] and histological subtype). This may have obscured important clinical heterogeneity in lymph node dissection. We plan to validate the consistency of efficacy across different staging and pathological types through expanded sample size and multicenter prospective studies, while further refining experimental methods.

## Data Availability

The original contributions presented in the study are included in the article/supplementary material. Further inquiries can be directed to the corresponding author.
